# Local and Systemic IKKε and NF-κB Signaling Associated with Sjögren's Syndrome Immunopathogenesis

**DOI:** 10.1155/2015/534648

**Published:** 2015-08-26

**Authors:** Weiqian Chen, Jin Lin, Heng Cao, Danyi Xu, Bei Xu, Liqin Xu, Lihuan Yue, Chuanyin Sun, Guolin Wu, Wenbin Qian

**Affiliations:** ^1^Department of Rheumatology, The First Affiliated Hospital, College of Medicine, Zhejiang University, Hangzhou, Zhejiang 310003, China; ^2^Department of Chinese Internal Medicine, The First Affiliated Hospital, College of Medicine, Zhejiang University, Hangzhou, Zhejiang 310003, China; ^3^Department of Hematology, The First Affiliated Hospital, College of Medicine, Zhejiang University, Hangzhou, Zhejiang 310003, China

## Abstract

The activated NF-*κ*B signaling pathway plays an important role in pathogenesis of primary Sjögren's syndrome (pSS). The inhibitor of *κ*B (I*κ*B) kinase (IKK) family such as IKK*α*, IKK*β*, IKK*γ*, and IKK*ε*, is required for this signaling. Our aim was to investigate the role of IKK*α*/*β*/*γ*/*ε* in patients with untreated pSS. In minor salivary glands from pSS patients, phosphorylated IKK*ε* (pIKK*ε*), pI*κ*B*α*, and pNF-*κ*B p65 (p-p65) were highly expressed in ductal epithelium and infiltrating mononuclear cells by immunohistochemistry, compared to healthy individuals. pIKK*α*/*β* and pIKK*γ* were both negative. And pIKK*ε* positively related to expression of p-p65. Furthermore, pIKK*ε* and p-p65 expression significantly correlated with biopsy focus score and overall disease activity. Meanwhile, in peripheral blood mononuclear cells from pSS patients, pIKK*ε*, total IKK*ε*, pIKK*α*/*β*, and p-p65 were significantly increased by western blot, compared to healthy controls. However, there was no difference in IKK*γ* and I*κ*B*α* between pSS patients and healthy individuals. These results demonstrated an abnormality of IKK*ε*, I*κ*B*α*, and NF-*κ*B in pSS, suggesting a potential target of treatment for pSS based on the downregulation of IKK*ε* expression and deregulation of NF-*κ*B pathway.

## 1. Introduction

Primary Sjögren's syndrome (pSS) is a chronic systemic inflammatory autoimmune disease characterized by keratoconjunctivitis sicca and xerostomia. The exocrine glands of the skin and urogenital, respiratory, and gastrointestinal tract may also be involved. Moreover, extraglandular involvement is common, such as synovitis, interstitial lung disease, neuropathy, renal disease, vasculitis, autoimmune cytopenia, and hypergammaglobulinemia [1]. However, the precise etiopathogenesis remains unclear.

Nuclear factor kappa B (NF-*κ*B) is a family of DNA-binding proteins that contribute to many cellular responses to stimuli, notably the immune response and inflammation, through their effects on the transcription of proinflammatory cytokines [2]. The NF-*κ*B pathway has been implicated in the development of several autoimmune diseases through its activation of proinflammatory and antiapoptotic pathways [2–5].

NF-*κ*B proteins form dimers, which canonically activate gene expression although some dimers play a regulatory role. The most prominent NF-*κ*B dimer is present in the cytosol in an inactive state bound to a natural inhibitor termed I*κ*B*α* (inhibitor of *κ*B). NF-*κ*B activation in response to proinflammatory signals is dependent on polyubiquitination that targets I*κ*B*α* for degradation, releasing NF-*κ*B dimers from the NF-*κ*B-I*κ*B*α* complex, followed by translocation to the nucleus and binding to *κ*B enhancer elements of target genes.

I*κ*B kinase (IKK) is a large molecular weight complex consisting of three subunits, IKK*α*, IKK*β*, and IKK*γ*. IKK*α* and IKK*β* (called canonical IKK) serve as catalytic subunits that phosphorylate I*κ*B*α* on two serine residues to activate the NF-*κ*B [6], while IKK*γ* (also called NEMO) is a regulatory subunit. I*κ*B kinase *ε* (IKK*ε*) is a noncanonical IKK homolog, which shares 33% and 31% amino acid identity with the corresponding domains in IKK*α* and IKK*β*, and plays an important role in activating NF-*κ*B signaling by phosphorylating I*κ*B*α* [6–8].

There is increasing evidence of correlation between NF-*κ*B signaling and the chronic inflammation that characterizes pSS. Villalon et al. [9] used immunohistochemical (IHC) analysis to find nuclear translocation of NF-*κ*B in focal infiltrated lymphocytes and in the acini epithelium adjacent to the infiltrates from minor salivary glands (MSG) of patients with pSS. In distal normal acini and ductal structures from the infiltrates, there was no nuclear translocation. Lisi et al. [10] reported that the gene and protein expression of I*κ*B*α* was decreased in pSS monocytes, and NF-*κ*B activity was increased, suggesting that the attenuated expression of I*κ*B*α* and resulting deregulation of NF-*κ*B may lead to pSS pathogenesis. Furthermore, NF-*κ*B p65 nuclear translocation has been induced in primary salivary epithelial cells from pSS patients by a variety of agents such as epidermal growth factor (EGF) and CD40 ligation [11, 12]. Finally, pSS autoantibodies can promote the activation of the NF-*κ*B pathway, leading to overexpression of multiple proangiogenic/proinflammatory factors. Inhibition of NF-*κ*B activity significantly abrogated the release of these cytokines [13].

The purpose of this study was to investigate whether the expression of IKK*α*/*β*/*γ*/*ε* is altered in MSG and peripheral blood mononuclear cells (PBMC) from untreated pSS patients. We try to find which IKK kinase may contribute to the pathogenesis of pSS and be suitable for potential targeted therapy.

## 2. Materials and Methods

### 2.1. Subjects

This cohort study included patients with untreated pSS from the Division of Rheumatology, The First Affiliated Hospital, College of Medicine, Zhejiang University. All pSS patients fulfilled the revised 2002 American-European criteria [14]. None of the pSS patients had evidence of connective tissue disorders, lymphoma, sarcoidosis, essential mixed cryoglobulinemia, or infection by hepatitis-B, hepatitis-C, or human immunodeficiency virus. This study was carried out between June 2013 and December 2014. Thirty-three patients had pSS, and 26 age- and sex-matched healthy individuals with complaints of dry mouth or eyes were studied in parallel as controls. Healthy individuals had no connective tissue disorders, neoplasms, or current infections. Patients with pSS were assessed for disease activity with the European League Against Rheumatism (EULAR) Sjögren's Syndrome Disease Activity Index (ESSDAI) scores [15]. Baseline characteristics of the 33 untreated pSS patients and 26 healthy controls are reported in Table 1. In the pSS group, female to male ratio was 15.5, and ESSDAI scores ranged from 2 to 37 points; the mean of ESSDAI scores was 13.5 ± 9.8, and the median was 8.0. One patient with systemic lupus erythematosus fulfilling the American College of Rheumatology revised classification criteria [16] was included as a disease control (see Supplemental Figure S4 in Supplementary Material available online at http://dx.doi.org/10.1155/2015/534648). All patients and controls did not receive glucocorticoids, immunosuppressants, or biological agents as therapy during the 6 months prior to inclusion in the study. All experimental protocols were approved by the ethics committee of The First Affiliated Hospital, College of Medicine, Zhejiang University. Patients and healthy volunteers were recruited after obtaining informed consent.

### 2.2. Clinical Features

Patient information on demographic data, clinical features, serological profile, and medications was obtained from medical records. Current pSS disease activity was measured using ESSDAI scores [15]. In all patients, ESSDAI scores were recorded, along with any incidence of constitutional symptoms (fever >37.5°C, night sweats, and/or involuntary weight loss of body weight >5% caused by disease itself), lymphadenopathy (lymphadenopathy ≥1 cm in any nodal region or ≥2 cm in inguinal region by ultrasound or splenomegaly), lymphoma (histologically diagnosed), glandular swelling (parotid, submandibular, or lachrymal swelling), arthritis (arthralgia in hands, wrists, ankles, and feet accompanied by morning stiffness or established synovitis), cutaneous involvement (current cutaneous vasculitis or purpura), lung involvement (interstitial lung disease shown by CT scan or X-ray with or without abnormal pulmonary function test), renal involvement (tubular acidosis, persistent proteinuria, glomerular involvement verified by renal biopsy, or renal failure), muscular involvement (myositis shown by electromyography or biopsy with weakness or elevated creatine kinase), peripheral neuropathy (verified by nerve conduction studies), central neuropathy (cranial nerve involvement of central origin, optic neuritis, or cerebral vasculitis), hematologic disorder (neutropenia, anemia, thrombocytopenia, or lymphopenia related to autoimmunity), and Raynaud's phenomenon. Anti-Ro/SSa and/or anti-La/SSb autoantibodies, erythrocyte sedimentation rate (ESR), rheumatoid factor (RF), complement components (C3 and C4), cryoglobulinemia, hypergammaglobulinemia (total IgG level >20 g/L), complete blood counts, and C-reactive protein (CRP) were measured.

### 2.3. MSG Histology and Patient Groupings

MSG (minor salivary gland) biopsy specimens were obtained with informed consent from 33 individuals undergoing diagnostic evaluation for sicca symptoms indicative of pSS and diagnosed as pSS by revised American-European SS consensus criteria [14]. The control group consisted of 26 gender-matched individuals with subjective complaints of dry mouth or eyes, who received an MSG biopsy but did not fulfill the criteria for pSS and had no histopathological evidence of pSS.

Biopsy specimens were fixed, embedded, sectioned (5 *μ*m), deparaffinized, rehydrated through alcohol, and stained with hematoxylin-eosin (HE). All pSS patients presented a biopsy focus score (FS) ≥1 (aggregate of at least 50 inflammatory cells per 4 mm^2^) [17], whereas the control group had FS <1. Germinal center (GC) formation was considered based on HE staining, defined as a well-circumscribed chronic inflammatory cell infiltrate consisting of at least 50 mononuclear cells exhibiting lymphoid organization, such as a densely packed dark zone and a light zone within otherwise normal salivary gland epithelium [18].

Histological grading was categorized in three groups based on inflammation as mild (pSS-I, focus score 1.0–2.0, *n* = 10), intermediate (pSS-II, focus score 2.1–3.0, *n* = 11), and advanced (pSS-III, focus score >3.0, *n* = 12). There were no significant differences between the three pSS subgroups in regard to sex and age. Compared to the mild group (pSS-I), the patients with pSS-II had longer disease duration, higher MSG biopsy score, and higher ESSDAI scores. Compared to the mild and intermediate groups (pSS-I + pSS-II), the patients with pSS-III represented a severe stage of disease, characterized by much longer disease duration, higher MSG biopsy score, many more clinical symptoms (constitutional symptoms, lymphadenopathy, glandular swelling, and lung involvement), higher ESR, highly activated B cells (RF and hypergammaglobulinemia), and much higher ESSDAI scores (Table 1).

### 2.4. MSG Immunohistochemistry

For immunohistochemical analysis, sections were processed for antigen retrieval with antigen unmasking solution (EGTA pH 8.0) before blocking by endogenous peroxidase with 1.5% H_2_O_2_ in 50% methanol for 10 minutes. Sections were incubated with blocking serum (goat or rabbit) for 30 minutes and incubated for 1 hour at 37°C with primary antibody. In this study, we used antibodies against phospho-IKK*ε* (clone D1B7, Rabbit IgG), IKK*ε* (clone D20G4, Rabbit IgG), phospho-IKK*α*/*β* (clone 16A6, Rabbit IgG), phospho-IKK*γ* (Rabbit IgG, catalogue number 2689), IKK*γ* (Rabbit IgG, catalogue number 2685), phospho-NF-*κ*B p65 (Rabbit IgG, catalogue number 3037), and NF-*κ*B p65 (clone E498, Rabbit IgG) all from Cell Signaling Technology, Boston, MA; IKK*α*/*β* (clone H470, rabbit polyclonal) from Santa Cruz Biotechnology, Santa Cruz, CA; I*κ*B*α* (clone E130, rabbit monoclonal) and phospho-I*κ*B*α* (clone EPR3148, rabbit monoclonal) from Abcam (Cambridge, MA). For control staining, primary antibodies were replaced with irrelevant isotype-matched antibodies (Jackson ImmunoResearch, West Grove, PA). Secondary antibody staining was developed using DAKO K5007 (DAKO, Glostrup, Denmark) for 30 minutes, followed by 3,3′-diaminobenzidine tetrahydrochloride substrate chromogen (DAKO, Glostrup, Denmark) for 30 minutes, and then counterstaining with Mayer's hematoxylin. Histopathology, ranking, and immunostaining were evaluated by a cytopathologist blind to diagnosis. To evaluate antigen expression, electronic images of 10 optical fields (×400 magnification) were taken across sections. The intensity of staining was evaluated semiquantitatively as follows: negative (−); weak, patchy (+); moderate, patchy [<50% of the cells] (++); moderate, diffuse [>50% of cells] (+++); strong, diffuse [>50% of cells] (++++) [19, 20]. Immunostaining of serial sections provided evidence of antigen coexpression.

### 2.5. Western Blot Analysis

Protein lysates obtained from equal numbers of PBMC from patients with pSS and healthy donors were subjected to sodium dodecyl sulfate-polyacrylamide gel electrophoresis (SDS-PAGE) according to standard electrophoresis and transfer techniques. Membranes were incubated for 90 min for detection of phospho-IKK*ε*, IKK*ε*, phospho-IKK*α*/*β*, phospho-IKK*γ*, IKK*γ*, phospho-NF-*κ*B p65, NF-*κ*B p65, IKK*α*/*β*, I*κ*B*α*, and phospho-I*κ*B*α* using the same antibodies that were used for IHC (see above). Secondary staining was done with secondary antibody-HRP conjugate (MultiSciences (Lianke) Biotech Co., Hangzhou, China), and blots were developed using the EZ-ECL chemiluminescence detection kit (Biological Industries, Kibbutz Beit Haemek, Israel). *β*-actin was used as protein loading control. Relative IKK protein expression was demonstrated as a ratio (IKK gray scale/*β*-actin gray scale) by Bio-Rad Quantity One software.

### 2.6. RNA Extraction, cDNA Synthesis, and Semiquantitative Real-Time PCR

PBMC from pSS patients and healthy individuals were preserved in TRIzol reagent (Life Technologies, Grand Island, NY) and stored at −80°C. Total RNA was extracted. cDNA was prepared from 1 *μ*g of RNA using oligo(dT) primers, dNTP, and SuperScript II reverse transcriptase (Life Technologies, Grand Island, NY). The resulting cDNA was amplified by real-time PCR using a BioRad CFX96 C1000 thermal cycler. Amplification was performed using SYBR Green expression assays for IKK*α* (forward: 5′-GCAGTAACCCCTCAGACATCAG-3′; reverse: 5′-GGGACAGTGAACAAGTGACAAC-3′), IKK*β* (forward: 5′-CAAGAGCCCAAGAGGAATCTC-3′; reverse: 5′-GGATGCTGGTTTTGAAGAAATC-3′), IKK*γ* (forward: 5′-GACCCCGCAGACTATCAATC-3′; reverse: 5′-CATCTCACACAGTTGGCTCTTC-3′), IKK*ε* (forward: 5′-CCGAGTTGCCTCTGTCTCTTTA-3′; reverse: 5′-GTGTTCTTAGCCTCCTGGTAGC-3′), I*κ*B*α* (forward: 5′-GGAGTTCACAGAGGACGAGC-3′; reverse: 5′-CTGGGGTCAGTCACTCGAAG-3′), and glyceraldehyde-3-phosphate dehydrogenase (GAPDH) (forward: 5′-GAAGGTGAAGGTCGGAGTC-3′; reverse: 5′-GAAGATAGGTGATGGGATTTC-3′) as a normalization control. The data were examined using the 2^−ΔΔCT^ method, and results are expressed as fold increase. Each sample was tested in triplicate, and tests were repeated three times.

### 2.7. Statistics

Correlation of ranked parameters was investigated by Spearman's correlation coefficient test, and comparisons between groups were performed using nonparametric Mann-Whitney test. For immunohistochemical data, pSS and healthy groups were compared by Pearson Chi-Square test. *P* < 0.05 was considered statistically significant. All analyses were performed using the statistical package SPSS 18.0 (SPSS, Chicago, IL).

## 3. Results 

### 3.1. Phosphorylated IKK*ε*, Phosphorylated I*κ*B*α*, and Phosphorylated NF-*κ*B p65 Were Highly Expressed in Ductal Epithelium and Infiltrating Mononuclear Cells of MSG in Patients with pSS

**Figure 1:**
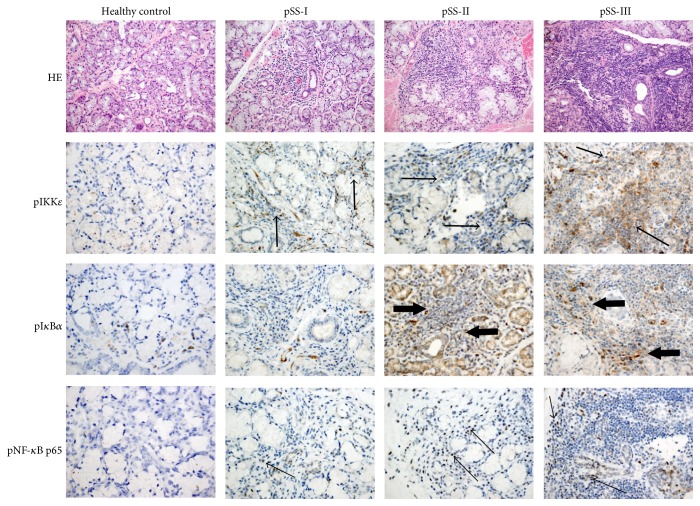
pIKK*ε*, pI*κ*B*α*, and pNF-*κ*B p65 were highly expressed in MSG from patients with pSS. Three pSS subgroups were classified by the grade of the inflammatory lesion (pSS-I: mild, pSS-II: intermediate, and pSS-III: severe lesions) by HE staining of MSG tissue (top row). The expression of pIKK*ε* (second row, middle arrow), pI*κ*B*α* (third row, large arrow), and pNF-*κ*B p65 (bottom row, small arrow) was also detected in sections of MSG tissue by immunohistochemical (IHC) staining (brown), combined with counterstaining with Mayer's hematoxylin (blue). More severe infiltration grade was accompanied by higher expression of pIKK*ε*, pI*κ*B*α*, and pNF-*κ*B p65. Representative examples are shown. Original magnification: ×200 for HE staining and ×400 for pIKK*ε*, pI*κ*B*α*, and pNF-*κ*B p65 IHC. All data are representative of three independent experiments.

First, we used immunohistochemical (IHC) staining to characterize the expression of proteins in the NF-*κ*B pathway in minor salivary gland biopsies from patients with pSS and normal controls. The goal of this aspect of the study was to see whether NF-*κ*B activation was related to severity of disease and to learn which cell types in the MSG tissue were sites of deregulated NF-*κ*B activity. IHC analysis revealed that phosphorylated IKK*ε* (pIKK*ε*) was highly expressed in ductal epithelium and infiltrating mononuclear cells, but not in the acinar epithelium of MSG from patients with pSS (Figure 1). Phosphorylated I*κ*B*α* was expressed in infiltrating mononuclear cells, ductal epithelium and acinar epithelium of MSG from patients with pSS (Figure 1). Total IKK*ε*, total IKK*α*/*β*, and total I*κ*B*α* were similarly expressed in ductal epithelium and mononuclear cells from pSS cases and in ductal epithelium from healthy controls. Total I*κ*B*α* was also expressed at similarly high levels in acinar epithelium of MSG from both patients with pSS and healthy controls (Supplementary Figure S1, Table 2). However, no expression of pIKK*α*/*β*, total IKK*γ*, or pIKK*γ* was found in this tissue (Supplementary Figure S2, Table 2). Phosphorylation of the NF-*κ*B family member p65 (p-p65), which indicates activation of NF-*κ*B signaling, was observed in the infiltrating mononuclear cells and ductal epithelium in 93.9% (31/33) of patients with pSS, but in none of the healthy controls (Figure 1, Table 2).

In total, pIKK*ε*, total IKK*ε*, total IKK*α*/*β*, pI*κ*B*α*, and p-p65 were more positively expressed in MSG from pSS patients compared to healthy individuals (*P* < 0.0001, *P* < 0.0001, *P* = 0.02, *P* = 0.003, and *P* < 0.0001, resp.) (Table 2). Furthermore, more severe infiltration grade in MSG was associated with higher expression of pIKK*ε*, pI*κ*B*α*, and pNF-*κ*B p65 (Figure 1). However, no increased trend of total IKK*α*/*β* expression was found in more advanced MSG lesions from pSS patients (data not shown).

Interestingly, pIKK*ε* was positively correlated to expression of p65 (*r* = 0.763, *P* < 0.0001). Furthermore, pIKK*ε* and p65 expression positively correlated with biopsy focus score (*r* = 0.656, *P* < 0.0001; *r* = 0.788, *P* < 0.0001, resp.), infiltration grade (*r* = 0.727, *P* < 0.0001; *r* = 0.818, *P* < 0.0001 resp.), and ESSDAI scores (*r* = 0.386, *P* = 0.027; *r* = 0.359, *P* = 0.040 resp.). Finally, pIKK*ε* and p-p65 expression was positively associated with RF (*r* = 0.321, *P* = 0.029; *r* = 0.355, *P* = 0.013, resp.), though no association with C3, C4, IgG, ESR, or CRP was seen.

### 3.2. Protein Expression of IKK*ε* and pNF-*κ*B p65 Was Significantly Increased in PBMC from Patients with pSS

**Figure 2:**
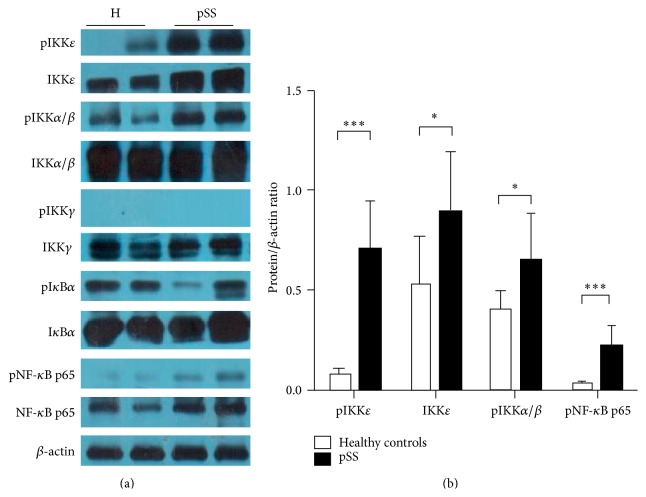
Protein expression of pIKK*ε*, total IKK*ε*, pIKK*α*/*β*, and pNF-*κ*B p65 were significantly increased in PBMC from patients with pSS, compared to healthy controls. (a) Phosphorylated and total protein expression of IKK*ε*, IKK*α*/*β*, IKK*γ*, I*κ*B*α*, and NF-*κ*B p65 in PBMC from two healthy controls (H) and two representative pSS patients was shown by western blot. (b) The relative expression of pIKK*ε*, total IKK*ε*, pIKK*α*/*β*, and pNF-*κ*B p65 were higher in pSS (*n* = 33), compared to healthy controls (*n* = 26). *β*-actin was used as endogenous control, and relative expression of each protein is shown as protein/*β*-actin ratio. ^*∗*^ *P* < 0.05;  ^*∗∗∗*^ *P* < 0.001. All data are representative of three independent experiments.

To learn more about the activity of the proinflammatory NF-*κ*B pathway in cases of pSS, we also looked at peripheral blood mononuclear cells to see if we could characterize NF-*κ*B activation in the white blood cells that mediate much of the autoimmunity. Using western blot of cell lysates, we saw that protein expression of pIKK*ε*, total IKK*ε*, and pNF-*κ*B p65 was significantly increased in PBMC from patients with pSS when compared to healthy controls (Figures 2(a) and 2(b)). Phosphorylation of IKK*α*/*β* was also enhanced in pSS (Figures 2(a) and 2(b)) while there was no difference in protein expression of total IKK*α*/*β*, pIKK*γ*, total IKK*γ*, pI*κ*B*α*, total I*κ*B*α*, or NF-*κ*B p65 between pSS patients and healthy individuals (Figure 2(a), Supplementary Figure S5A, 5B). Untreated Jurkat T leukemia cells served as a negative control for pIKK*ε* (Supplementary Figure S3), as reported previously [8]. The sizes of the proteins in question can be seen on the uncropped blots shown in Supplementary Figure S4.

Furthermore, there was a strong correlation between IHC staining and Western blot analyses. IHC staining of pIKK*ε* in MSG from pSS patients positively correlated with pIKK*ε* levels in PBMC lysates as measured by western blot (*r* = 0.843, *P* < 0.0001), and consistent results were also found for other proteins such as total IKK*ε* (*r* = 0.501, *P* = 0.004), pI*κ*B*α* (*r* = 0.468, *P* = 0.009), and p-p65 (*r* = 0.741, *P* < 0.0001).

### 3.3. No Abnormal mRNA Expression of IKK*α*/*β*/*γ*/*ε* and I*κ*B*α* Was Found in PBMC from Patients with pSS

To further explore NF-*κ*B activation in immune cells of pSS patients, we investigated whether the increased levels of pIKK*ε*, total IKK*ε*, or pIKK*α*/*β* protein were accompanied by enhanced RNA expression of these genes. However, as measured by quantitative real-time RT-PCR, the relative expression of IKK*ε* mRNA in PBMC from pSS patients was 1.19 ± 0.61-fold higher than the expression in PBMC from healthy individuals, with no significant difference between the groups (*P* = 0.12). Unsurprisingly, there were also no significant results for RNA levels of IKK*α* (1.08 ± 0.77-fold change, *P* = 0.60), IKK*β* (1.01 ± 0.55-fold change, *P* = 0.92), IKK*γ* (0.91 ± 0.32-fold change, *P* = 0.20), and I*κ*B*α* (0.96 ± 0.42-fold change, *P* = 0.63).

## 4. Discussion

Enhanced activation of NF-*κ*B has been described in minor salivary glands and cultured primary salivary epithelial cells from pSS patients [9–13]. However, it is still unclear what roles NF-*κ*B and related molecules play in the pathogenesis of pSS. In this study, we have identified that NF-*κ*B p65 is highly phosphorylated in minor salivary glands and PBMC from patients with pSS. We also saw that phosphorylation of NF-*κ*B p65 positively correlated with infiltration grade, biopsy focus score, and ESSDAI scores. Our data further supports the importance of NF-*κ*B activation in pSS. Furthermore, enhanced phosphorylation of pNF-*κ*B p65 is accompanied by phosphorylation of IKK*ε* and I*κ*B*α*, all of which are positively correlated with more severe infiltration grade in salivary glands and more severe disease. These results demonstrate aberrant IKK*ε*, I*κ*B*α*, and NF-*κ*B signaling in pSS tissue, which may partially explain the immunologic etiology of pSS.

We also found that phosphorylated IKK*α*/*β* was at slightly higher levels in PBMC from patients with pSS, and IKK*α*/*β* was detected at higher levels in MSG tissue from these patients when compared to controls. However, within the pSS patient population, the level of total IKK*α*/*β* did not correlate with disease activity severity (data was not shown). And phosphorylated IKK*α*/*β*, phosphorylated IKK*γ*, and total IKK*γ* were also undetectable in MSG from pSS. This suggested that if IKK-mediated NF-*κ*B signaling is related to pathogenesis of pSS, it is more dependent on noncanonical IKK*ε* rather than canonical IKK*α*/*β*.

We also investigated whether the significantly abnormal protein levels of IKK in pSS specimens, as detected by IHC staining and western blot, were accompanied by similar changes of IKK mRNA in PBMC. However, no abnormal mRNA expression of IKK*α*/*β*/*γ*/*ε* or I*κ*B*α* was found in PBMC from patients with pSS. These results suggest that the deregulation of NF-*κ*B activity is the result of altered translation, stability, and/or phosphorylation of proteins rather than upregulation of the genes that encode proteins such as IKK*ε*, I*κ*B*α*, or NF-*κ*B p65.

Constitutive IKK*ε* expression is only observed in specific cell types (e.g., peripheral blood leukocytes) and tissues such as pancreas, thymus, and spleen [21]. However, in other cell types (e.g., fibroblasts), IKK*ε* is rapidly upregulated by proinflammatory cytokines, microbial products (such as LPS and double-stranded RNA), and phorbol esters. Therefore, it has also been called inducible IKK [21, 22]. Since NF-*κ*B mediates a number of physiological functions, nonselective and complete inhibition of the NF-*κ*B signal pathway may lead to serious side-effects. There is progress in the development of IKK*α*/*β* inhibitors as novel anti-inflammatory agents, but so far with limited success; IKK*β* appears to be a more tractable target for inhibition than IKK*α*, but IKK*β* inhibitors produce more cellular and systemic toxicity [23]. IKK*ε* knockout mice, in contrast to IKK*α* or IKK*β* knockout mice, are viable and fertile [24]. Therefore, IKK*ε* may be a potential target for the treatment of autoimmunity with fewer side-effects.

Recently, there has been rapid progress in uncovering the function and mechanism of action of IKK*ε* in immune signaling. IKK*ε* has been associated with the pathology of autoimmune diseases such as rheumatoid arthritis (RA), systemic lupus erythematosus (SLE), and psoriasis. For instance, IKK*ε* is constitutively expressed in synovium and phosphorylated in synovial intimal lining of RA patients, resulting in uncontrolled IRF3-driven production of proinflammatory mediators such as IFN-*β* and chemokines [25]. Further supporting a major contribution of IKK*ε* to the pathogenesis of RA is the finding that mice deficient in IKK*ε* show less synovial inflammation and modestly decreased clinical arthritis in a passive K/B × N arthritis model due to lower expression of inflammatory mediators [26]. Also, single nucleotide polymorphisms (SNPs) in IKBKE (the gene encoding IKK*ε*) have been found to be linked to RA [27], and a candidate gene study implicated* IKBKE* as a susceptibility locus for SLE [28]. Another study showed an association between* IKBKE* SNPs and antibody-positive pSS in a Scandinavian cohort, although this was not seen in a United Kingdom cohort [29]. Finally, IKK*ε* has been identified as a potential therapeutic target in psoriasis using a computational approach, combining microarray analysis and protein-protein interaction prediction [30]. Our results are in agreement with these studies in suggesting that IKK*ε*-mediated NF-*κ*B signaling may relate to pathogenesis of pSS.

In minor salivary gland lesions, the mononuclear cells infiltrate mainly consists of T and B cells as well as some macrophages [31, 32]. Along with these cells producing antibodies and cytokines in the vicinity of the lesions, highly activated B lymphocytes are a feature of the peripheral circulation of pSS patients, contributing to the presence of hypergammaglobulinemia and anti-SSA/SSB antibodies [33]. It has been reported that anti-SSA/SSB antibodies can activate NF-*κ*B in the salivary epithelial cells, leading to overexpression of multiple proangiogenic/proinflammatory cytokines [13]. Therefore, we hypothesize that local and systemic immunoreactions in MSG or peripheral circulation will trigger release of proinflammatory cytokines and autoantibody, activating IKK*ε*-mediated NF-*κ*B signaling, contributing to the expression of inflammatory genes and creating a vicious circle of chronic inflammation.

Striking abnormalities in the NF-*κ*B signal transduction pathway, involving phosphorylation of I*κ*B*α*, have been observed in several autoimmune diseases [34–36]. However, in our study, compared to healthy controls, mRNA and protein expression of I*κ*B*α* was almost normal in PBMC from pSS. Lisi et al. reported that the gene and protein expression of I*κ*B*α* was decreased in pSS CD14^+^ monocytes [10]. Although our results appear inconsistent with the results of that study, this may be explained by the fact that Lisi et al. focused on CD14^+^ monocytes, not PBMC. However, we and Lisi et al. both found that NF-*κ*B activity was increased. Furthermore, we did find that the I*κ*B*α* was phosphorylated in MSG from patients with pSS and that a more severe infiltration grade in MSG was associated with a higher expression of pI*κ*B*α*.

Our study has strengths and limitations. One reason our results are robust is that our patients with pSS had not received glucocorticoids or other immunosuppressants during the 6 months prior to inclusion in the study, thus avoiding the effect of immunosuppressants on IKK-related proteins. Our study population was well characterized and we believe the healthy control group was appropriately chosen and matched with the group of pSS patients. However, our results could be strengthened by a greater sample size or by including other groups such as pSS patients after treatment or disease controls such as lupus patients and secondary SS. Secondly, we have not yet cultured the salivary gland epithelial cells (SGEC) from the MSG obtained from pSS patients or studied the expression of IKK/I*κ*B*α*/NF-*κ*B in SGEC under different inflammatory conditions in vitro. Finally, further studies are needed to investigate the clinical role and mechanism of IKK*ε* in pSS animal models, particularly studies using IKK*ε*-deficient animals and studies on whether IKK*ε* antagonist treatment can reverse the inflammation and resulting normal function of salivary glands in a pSS animal model.

## 5. Conclusions

Our data sheds light on the role of IKK*ε*, I*κ*B*α*, and NF-*κ*B in primary Sjögren's syndrome. The present study suggests that IKK*ε*/I*κ*B*α*/NF-*κ*B signaling pathway may play an important role in the pathogenesis of pSS. We suggest that IKK*ε* may be a potential target for the treatment of pSS: by attenuating expression or activation of IKK*ε*, it may be possible to repress the downstream signaling that produces the deregulation of NF-*κ*B pathways.

## Supplementary Material

Supplementary Figure 1: Expression of total IKKε, total IKKα/β and total IκBα protein was shown in MSG from patients with pSS and healthy controls by IHC analysis.
Supplementary Figure 2: Expression of pIKKα/β, pIKKγ and total IKKγ was not seen in MSG tissues from patients with pSS and healthy controls by IHC analysis.
Supplementary Figure 3: Untreated Jurkat T leukemia cells were served as a negative control for pIKKε.
Supplementary Figure 4: Full images of western blots stained for phosphorylated and total protein expression of IKKε, IKKα/β, IKKγ, IκBα and NF-κB p65 in PBMC from patients with pSS, SLE, and healthy controls.
Supplementary Figure 5: Relative expression of total IKKα/β, pIKKγ, total IKKγ, pIκBα, total IκBα and total NF-κB p65 were similar in PBMC from healthy controls and patients with pSS.

## Figures and Tables

**Table 1:** Characteristics of the pSS patients included in the study.

Feature		pSS total (*n* = 33)	pSS-I (*n* = 10)	pSS-II (*n* = 11)	pSS-III (*n* = 12)	Controls (*n* = 26)
General	Age, mean ± SD years	50.6 ± 8.8	48.6 ± 6.8	49.8 ± 10.6	52.9 ± 8.0	52.2 ± 7.3
	Female/male	31/2	9/1	10/1	12/0	25/1
	Disease duration, mean ± SD months	21.0 ± 29.2	8.2 ± 4.5	19.5 ± 18.9^*∗*^	42.2 ± 40.0^*∗*^	NA

Histological (MSG biopsy)	Biopsy focus score (number of lymphocytic foci/4 mm^2^), mean ± SD, median (range)	2.9 ± 1.7 2.6 (1.0–6.4)	1.1 ± 0.1 1.1 (1.0–1.3)	2.5 ± 0.4^*∗∗∗*^ 2.6 (2.1–3.0)	4.8 ± 1.1^*∗∗∗*^ 5.0 (3.1–6.4)	0
	Germinal centers (GC) (+/−)	3/30	0/10	1/10	2/10	0
	Constitutional symptoms (+/−)	12/21	1/9	2/9	9/3^*∗∗∗*^	0

Clinical	Lymphadenopathy (+/−)	17/16	3/7	3/8	11/1^*∗∗∗*^	0
	Lymphoma (+/−)	0/33	0/10	0/11	0/12	0
	Glandular swelling (+/−)	9/24	1/9	1/10	7/5^*∗∗*^	0
	Arthritis (+/−)	11/22	5/5	4/7	2/10	3/23
	Cutaneous involvement (+/−)	5/28	0/10	2/9	3/9	0
	Lung involvement (+/−)	6/27	1/9	1/10	4/8^*∗*^	0
	Renal involvement (+/−)	2/31	0/10	1/10	1/11	0
	Muscular involvement (+/−)	2/31	0/10	0/11	2/10	0
	Peripheral neuropathy (+/−)	3/30	0/10	1/10	2/10	0
	Central neuropathy (+/−)	3/30	0/10	1/10	2/10	0
	Hematologic disorder (+/−)	15/18	5/5	6/5	4/8	2/24
	Raynaud's phenomenon (+/−)	3/30	0/10	1/11	2/10	NA

Laboratory	Anti-SSa (%)	87.9	80.0	81.8	100.0	11.5
	Anti-SSb (%)	36.3	20.0	36.3	50.0	3.8
	ESR, mean ± SD mm/h	40.6 ± 27.8	33.6 ± 28.2	39.0 ± 26.2	48.4 ± 25.4^*∗*^	19.2 ± 15.1
	RF, mean ± SD U/L	238.4 ± 294.9	101.4 ± 246.7	204.4 ± 253.3	375.0 ± 320.8^*∗∗*^	25.1 ± 25.2
	C3, mean ± SD mg/dL	99.3 ± 37.0	75.71 ± 40.3	89.6 ± 42.3	123.8 ± 19.6	96.6 ± 33.4
	C4, mean ± SD mg/dL	18.8 ± 9.8	14.3 ± 8.1	14.4 ± 11.2	13.8 ± 7.3	20.3 ± 10.1
	Cryoglobulinemia (%)	3.0	0	0	1/11	0
	Hypergammaglobulinemia (%)	57.6	4/6	4/7	11/1^*∗∗*^	0
	ESSDAI scores, mean ± SD	13.5 ± 9.8	5.5 ± 2.9	9.5 ± 5.4^*∗*^	23.7 ± 7.6^*∗∗∗*^	NA

pSS-I, pSS-II, and pSS-III: pSS patients with mild, intermediate, and advanced MSG lesions based on histological grading, respectively; ESR: normal range 0–20 mm/h, RF: normal range 0–20 U/L, C3: normal range 75–140 mg/dL, C4: normal range 10–40 mg/dL, hypergammaglobulinemia: IgG level > 20 g/L, and NA: not applicable. Statistical analysis was done between patients with pSS-II and patients with pSS-I, or patients with pSS-III and patients with pSS-II or pSS-I (pSS-II + pSS-I). ^*∗*^ *P* < 0.05, ^*∗∗*^ *P* < 0.01, and ^*∗∗∗*^ *P* < 0.001.

**Table 2:** IKK*α*/*β*/*γ*/*ε* expression in the MSG from pSS patients and healthy controls by immunohistochemistry.

	Number	pIKK*ε*	IKK*ε*	pIKK*α*/*β*	IKK*α*/*β*	pIKK*γ*	pI*κ*B*α*	I*κ*B*α*	p-p65
		Cytoplasm positive	Cytoplasm positive	Cytoplasm positive	Cytoplasm positive	Cytoplasm positive	Cytoplasm positive	Cytoplasm positive	Nuclear positive
Primary Sjögren syndrome	33	−: 0 +: 8 ++: 14 +++: 11 ++++: 0	−: 0 +: 4 ++: 8 +++: 15 ++++: 6	−: 33	−: 5 +: 6 ++: 5 +++: 14 ++++: 3	−: 33	−: 13 +: 5 ++: 7 +++: 8 ++++: 0	−: 0 +: 7 ++: 6 +++: 17 ++++: 3	−: 2 +: 16 ++: 15 +++: 0 ++++: 0

Healthy controls	26	−: 23 +: 3 ++: 0 +++: 0 ++++: 0	−: 6 +: 12 ++: 3 +++: 5 ++++: 0	−: 26	−: 4 +: 5 ++: 13 +++: 4 ++++: 0	−: 26	−: 22 +: 2 ++: 2 +++: 0 ++++: 0	−: 1 +: 4 ++: 8 +++: 12 ++++: 1	−: 26

The intensity of staining was evaluated semiquantitatively as follows: negative (−); weak, patchy (+); moderate, patchy [<50% of the cells] (++); moderate, diffuse [>50% of cells] (+++); strong, diffuse [>50% of cells] (++++).
