# Supplementary material for: Local and Systemic IKKε and NF-κB Signaling Associated with Sjögren's Syndrome Immunopathogenesis
Source: J Immunol Res. 2015 Aug 26;2015:534648. doi: 10.1155/2015/534648 (PMC4563092; doi:10.1155/2015/534648)
Supplement: Supplementary file 1 — Supplementary Figure 1: Expression of total IKKε, total IKKα/β and total IκBα protein was shown in MSG from patients with pSS and healthy controls by IHC analysis. Supplementary Figure 2: Expression of pIKKα/β, pIKKγ and total IKKγ was not seen in MSG tissues from patients with pSS and healthy controls by IHC analysis. Supplementary Figure 3: Untreated Jurkat T leukemia cells were served as a negative control for pIKKε. Supplementary Figure 4: Full images of western blots stained for phosphorylated and total protein expression of IKKε, IKKα/β, IKKγ, IκBα and NF-κB p65 in PBMC from patients with pSS, SLE, and healthy controls. Supplementary Figure 5: Relative expression of total IKKα/β, pIKKγ, total IKKγ, pIκBα, total IκBα and total NF-κB p65 were similar in PBMC from healthy controls and patients with pSS. [file 534648.f1.pdf]

Local and Systemic IKKε and NF-κB-signaling Associated with Sjögren's Syndrome Immunopathogenesis

Weiqian Chen[1], Jin Lin[1, *], Heng Cao[1], Danyi Xu[1], Bei Xu[1], Liqin Xu[1], Lihuan Yue[1], Chuanyin Sun[1], Guolin Wu[2], Wenbin Qian[3, *]

Supplemental Information

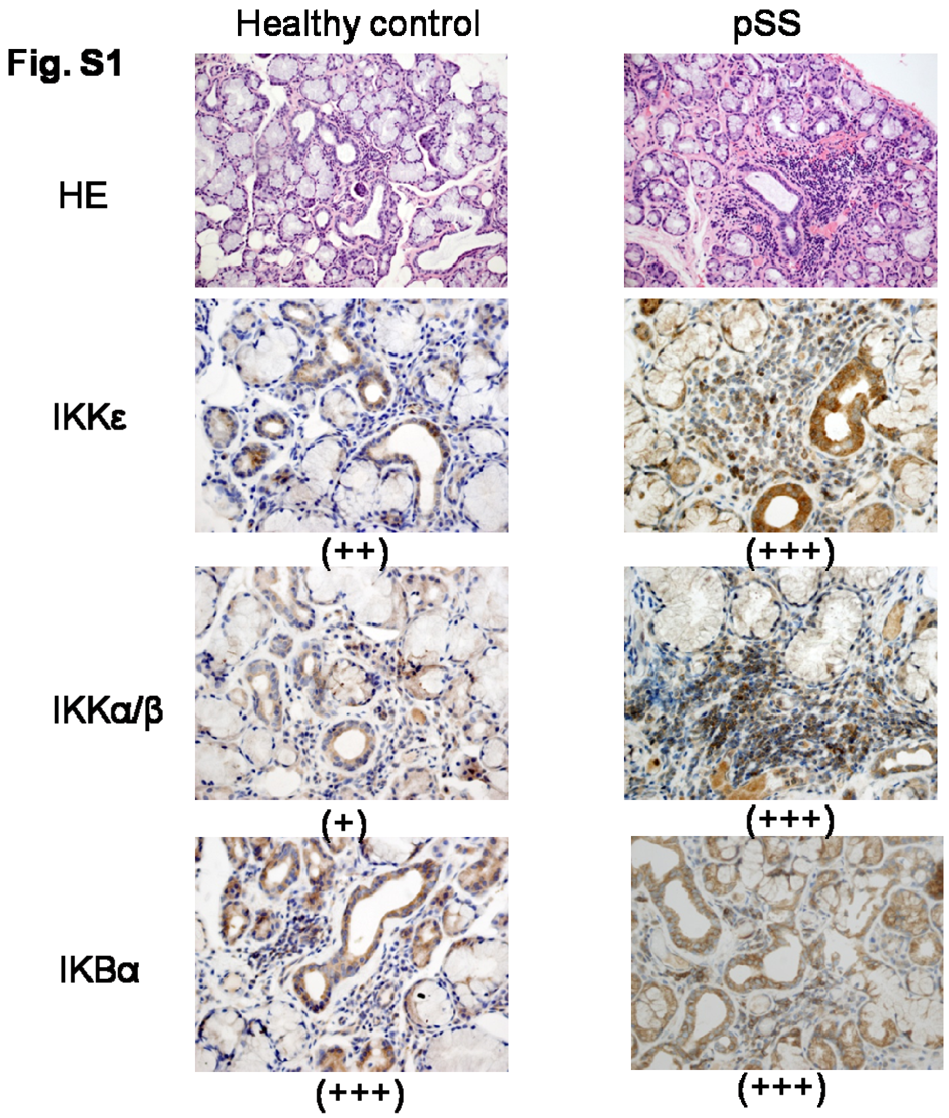

Figure S1. Expression of total IKKε, total IKKα/β and total IKBα protein in minor salivary glands (MSG) from patients with primary Sjögren's syndrome (pSS) and healthy controls. Sections of MSG tissue were stained with HE (top row) to observe

typical morphology of the ductal epithelium, acinar epithelium and infiltrating cells in MSG tissues. These sections were also stained for expression of total IKKε (second row), total IKKα/β (third row) and total IKBα (bottom row), and observed by immunohistochemical (IHC) analysis (brown), combined with counterstaining with Mayer’s hematoxylin (blue). Representative examples are shown in patients with pSS and normal control. Original magnification: ×200 for HE staining, ×400 for total IKKε, IKKα/β and IKBα IHC.

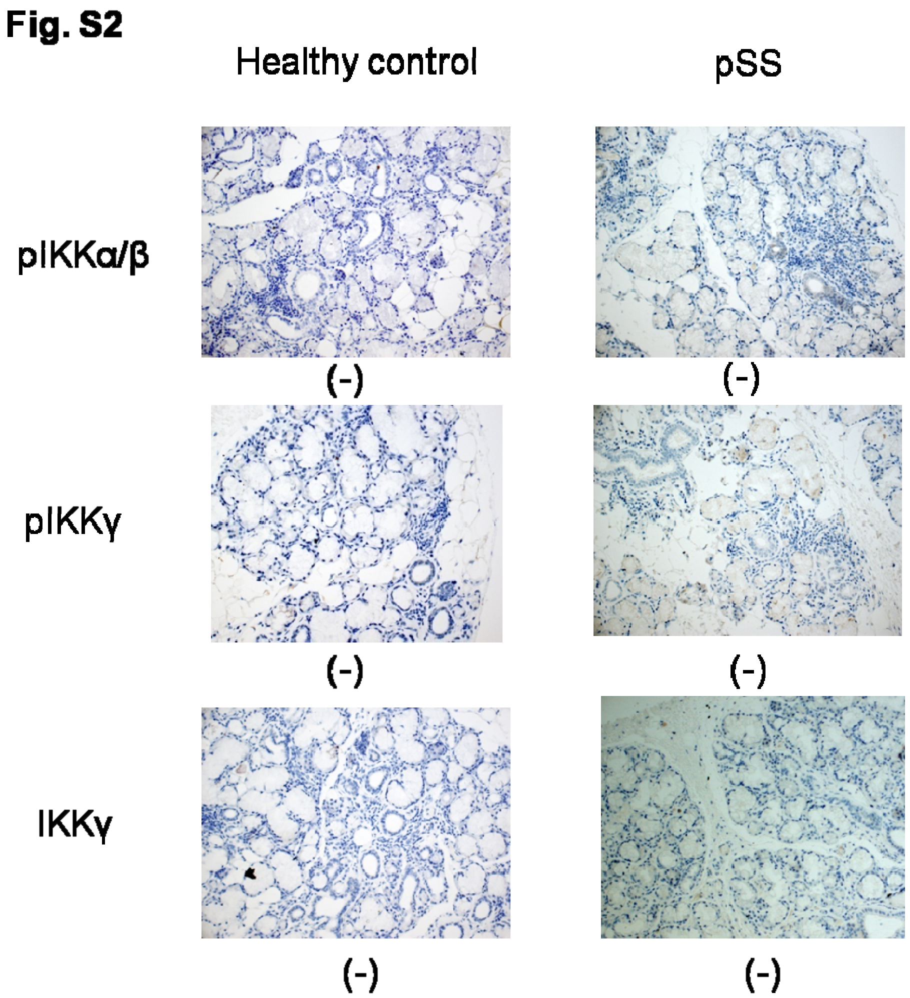

Figure S2. Expression of pIKKα/β, pIKKγ and total IKKγ was not seen in MSG tissues by IHC analysis. Staining was performed as in Figure S1, using antibodies against phosphorylated IKKα/β (top row), phosphorylated IKKγ (second row), and

total IKKγ (bottom row), combined with counterstaining with Mayer's hematoxylin (blue). Representative examples are shown from patients with pSS and healthy controls. Original magnification: ×200 for pIKKα/β, pIKKγ and total IKKγ IHC.

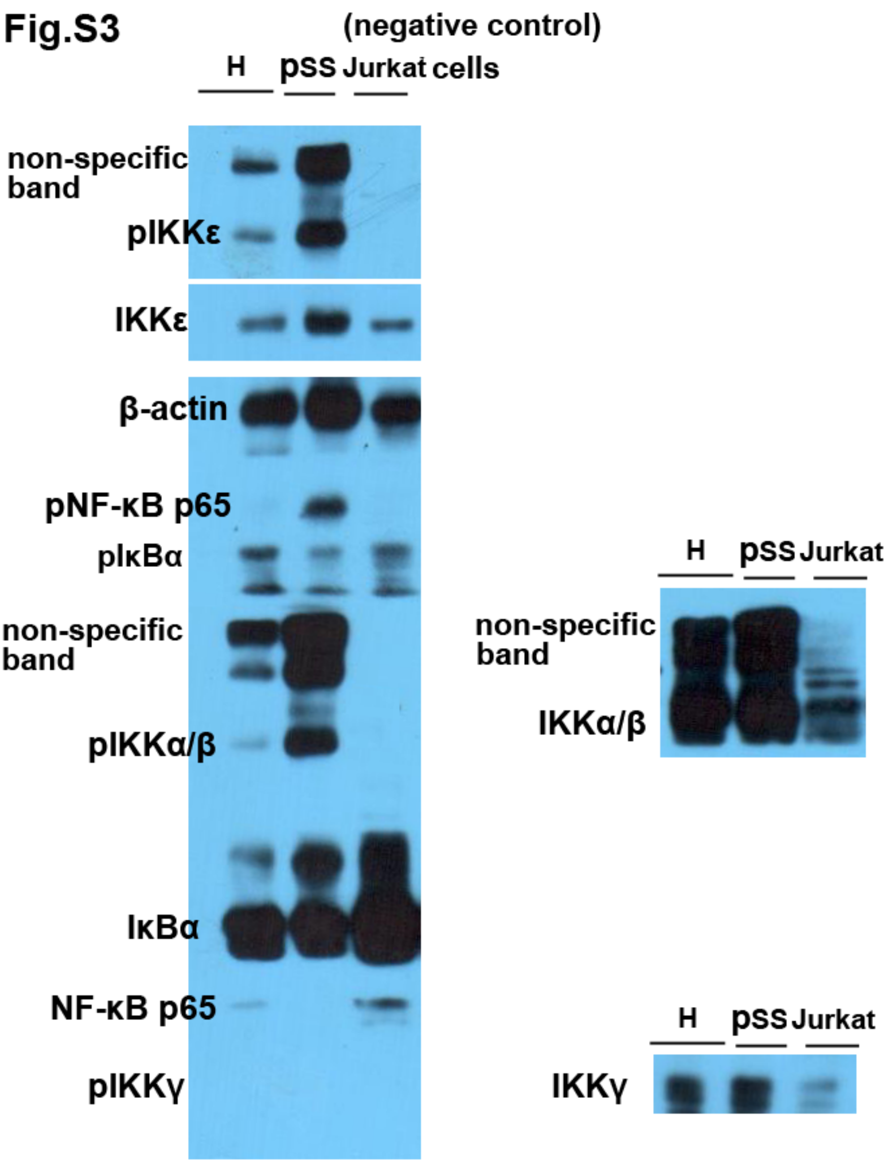

Figure S3. Protein expression of pIKKε, total IKKε and pNF-κB p65 was significantly increased in PBMC from patients with pSS. Peripheral blood mononuclear cells (PBMC) from patients with primary Sjögren's syndrome (pSS) and healthy individuals (H) were lysed and assayed for phosphorylated and total protein

expression of IKKε, IKKα/β, IKKγ, IκBα and NF-κB p65 using western blot. Untreated Jurkat T leukemia cells, obtained from the Cell Bank of the Chinese Academy of Sciences (Shanghai, China) served as a negative control. β-actin served as loading control for normalization. All data are representative of three repeated experiments.

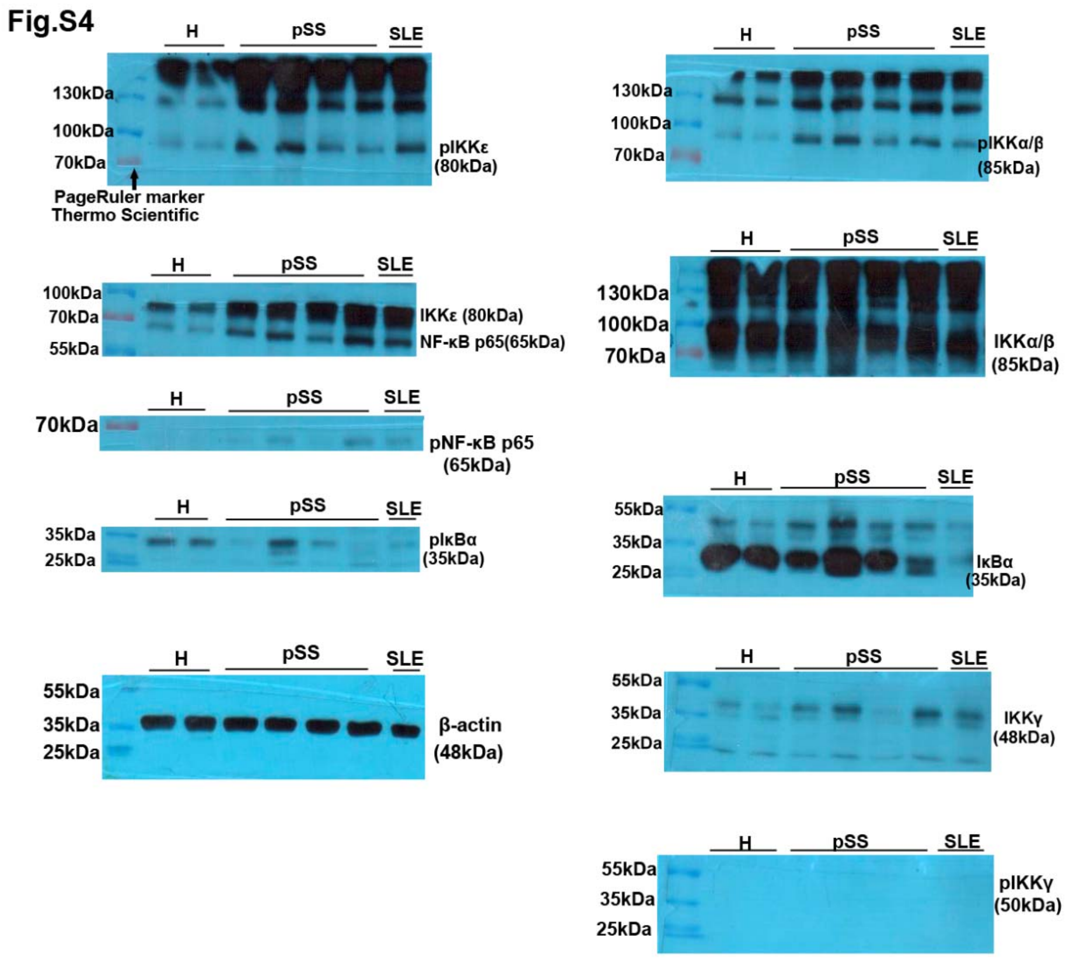

Figure S4. Full images of western blots stained for phosphorylated and total protein expression of IKKε, IKKα/β, IKKγ, IκBα and NF-κB p65 in PBMC. Peripheral blood

mononuclear cells (PBMC) from two healthy controls (H), four representative pSS patients and one patient with systemic lupus erythematosus (SLE) were lysed and analyzed for protein expression by western blot as in Figure S3. In all blots, Thermo Scientific PageRuler Plus Protein Ladder served as protein size standards (red band: 70kDa) and β-actin served as protein control. All data are representative of three repeated experiments.

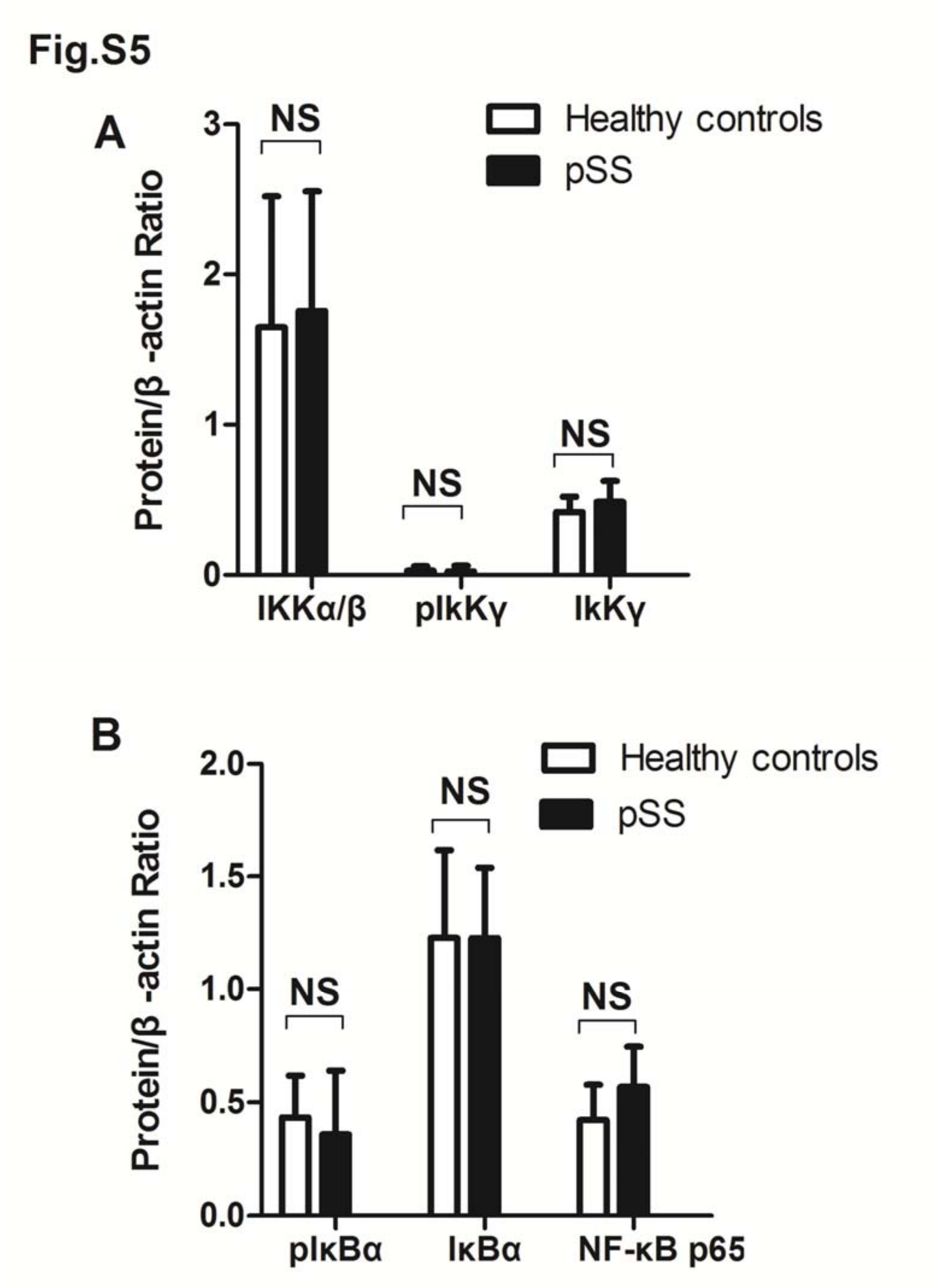

Figure S5. Relative expression of total IKKα/β, pIKKγ, total IKKγ, pIKBα, total IKBα and total NF-κB p65 were similar in PBMC from healthy controls and patients with pSS. Peripheral blood mononuclear cells (PBMC) from healthy controls (n=26) and pSS patients (n=33) were lysed and analyzed for protein expression by western blot as in Figure S3. For each protein, relative expression was quantified as the ratio of the protein to β-actin. NS: not significant.
